# Commentary: Management of non-palpable subcentimeter testicular incidentalomas

**DOI:** 10.3389/fonc.2025.1622614

**Published:** 2025-06-02

**Authors:** Jia You, Gang Li

**Affiliations:** Department of Urology, Wuhan Children’s Hospital (Wuhan Maternal and Child Healthcare Hospital), Tongji Medical College, Huazhong University of Science and Technology, Wuhan, Hubei, China

**Keywords:** testicular tumor, incidentalomas, subcentimeter, non-palpable, growth rate, management

Testicular tumors represent a significant pathological concern within the male reproductive system, with incidence rates exhibiting a consistent annual increase. Extensive evidence indicates a correlation between tumor size and malignant potential; lesions measuring ≥5 cm in diameter often indicate malignancy and require radical orchiectomy (1, 2). However, the clinical management of non-palpable, incidental testicular tumors measuring <10 mm remains debated, as approximately 30% are malignant (3). These asymptomatic tumors are frequently identified incidentally during ultrasonography and often associated with negative tumor markers, complicating diagnosis and potentially leading to unnecessary surgery or delayed treatment.

The recent study conducted by Bertolotto et al. (4) explores the link between growth rates and the nature of testicular incidentalomas under 10 mm, provides valuable insights into the correlation between quantitative growth kinetics and histopathological outcomes. It reveals that growth rates are crucial for distinguishing malignant from non-malignant lesions, with a size increase over 1 mm within 3 months or more than 2 mm within 6 months strongly indicating malignancy (sensitivity >90%). The study supports active monitoring of these small incidentalomas using serial high-frequency ultrasonographic rather than immediate surgical intervention, especially in younger patients, to minimize the risk of losing testicular function.

## Discussion

Determining whether testicular tumors are benign or malignant before surgery is challenging and crucial for treatment decisions. Key diagnostic tools include serum tumor markers, ultrasound, and clinical features like age and symptoms. Recent studies highlight that smaller tumors are usually benign, with most small incidental lesions being non-neoplastic. Bieniek et al. (5) found only six malignant cases among 120 subcentimeter incidental lesions, all over 5 mm. Scandura et al. (6) reported two-thirds of lesions under 5 mm were benign. A recent systematic review showed about 87% of lesions smaller than 5 mm were non-malignant (3).

Despite this, balancing early detection and avoiding overtreatment is essential, as small lesions can be malignant. Malignant tumors typically grow rapidly, unlike benign or non-neoplastic lesions, which grow more slowly. The growth rate thresholds proposed by Bertolotto et al. (4), corroborated by histopathological evidence, to guide clinical decisions. Their research indicates that conservative management can avoid surgery in 70% of cases.

However, several limitations warrant consideration in this study. Firstly, only 39 of 130 lesions were histopathologically verified, with a 46% malignancy rate, and 88 lesions remained stable, indicating possible selection bias. Secondly, growth overlap in 2 of 12 non-neoplastic lesions suggests the need to consider imaging features like vascularity and calcifications for better diagnosis. Additionally, non-standardized ultrasound intervals, such as 3-month versus 6-month can impact growth rate accuracy, especially for slow-growing tumors like teratomas.

Managing testicular incidentalomas involves addressing the limitations of surveillance strategies, with patient adherence being crucial for effective monitoring. Clear communication and collaborative decision-making between clinicians and patients are essential, and strategies like personalized schedules and reminders can enhance adherence. The psychological impact of continuous monitoring, which may cause anxiety, should be addressed through psychological support. Additionally, the cost-effectiveness of routine imaging, such as ultrasonography, must be carefully evaluated to balance costs and benefits. Healthcare providers must evaluate the economic impact on individual patients and overall resource allocation.

## Conclusion

Implementing a structured follow-up protocol for non-palpable subcentimeter testicular incidentalomas has important implications for urologists, radiologists, and patients. Urologists benefit from a standardized surveillance protocol that guides clinical decisions and reduces unnecessary interventions. Radiologists ensure accurate imaging evaluations for early detection of changes. Clear communication with patients about the process, risks, and benefits reduces anxiety and improves adherence. By integrating these perspectives, healthcare providers can create a patient-centered approach that improves outcomes and resource utilization in managing testicular incidentalomas.
